# Murine 5T multiple myeloma cells induce angiogenesis *in vitro* and *in vivo*

**DOI:** 10.1038/sj.bjc.6600137

**Published:** 2002-03-04

**Authors:** E Van Valckenborgh, H De Raeve, L Devy, S Blacher, C Munaut, A Noël, E Van Marck, I Van Riet, B Van Camp, K Vanderkerken

**Affiliations:** Department of Haematology and Immunology, Free University Brussels, Laarbeeklaan 103, B-1090 Brussels, Belgium; Department of Pathology, University Hospital Antwerp, Wilrijkstraat 10, B-2650 Edegem, Belgium; Laboratory of Tumor and Developmental Biology, University of Liège, Tour de Pathology (B23), Sart-Tilman, B-4000 Liège, Belgium

**Keywords:** multiple myeloma, angiogenesis, rat aortic ring assay, MVD

## Abstract

Multiple myeloma is a B cell malignancy. Recently, it has been demonstrated that bone marrow samples of patients with multiple myeloma display an enhanced angiogenesis. The mechanisms involved seem to be multiple and complex. We here demonstrate that the murine 5T multiple myeloma models are able to induce angiogenesis *in vitro* b*y* using a rat aortic ring assay and *in vivo* by determining the microvessel density. The rat aortic rings cultured in 5T multiple myeloma conditioned medium exhibit a higher number of longer and more branched microvessels than the rings cultured in control medium. In bone marrow samples from 5T multiple myeloma diseased mice, a statistically significant increase of the microvessel density was observed when compared to bone marrow samples from age-matched controls. The angiogenic phenotype of both 5T multiple myeloma cells could be related, at least in part, to their capacity to produce vascular endothelial growth factor. These data clearly demonstrate that the 5T multiple myeloma models are good models to study angiogenesis in multiple myeloma and will allow to unravel the mechanisms of neovascularisation, as well as to test new putative inhibitors of angiogenesis.

*British Journal of Cancer* (2002) **86**, 796–802. DOI: 10.1038/sj/bjc/6600137 www.bjcancer.com

© 2002 Cancer Research UK

## 

Multiple myeloma (MM) is a B cell neoplasm with three major characteristics: the expansion of malignant plasma cells mainly localised in the bone marrow, the presence of a monoclonal serum immunoglobulin and the activation of osteoclasts leading to osteolysis. The disease occurs mainly at older age and is still incurable in spite of the progress in treatment. Recently, it has been demonstrated that angiogenesis or neovascularisation is also involved in MM disease and its extent, as measured by microvessel density (MVD) of the bone marrow, correlated well with the activity of MM (Vacca *et al*, 1994). The human U266 MM cell line and bone marrow plasma cells from patients with active MM display a strong angiogenic potential (Vacca *et al*, 1998, 1999; Laroche *et al*, 2001). Moreover, bone marrow MVD also seems to be a prognostic factor of survival in MM patients (Sezer *et al*, 2000).

Angiogenesis is a complex process in which new blood vessels develop from pre-existing ones. It is controlled by a balance between pro-angiogenic and anti-angiogenic factors (Talks and Harris, 2000). While this balance is highly regulated in physiological circumstances such as reproduction, development and wound repair, an imbalance is associated with a variety of disorders including cancers (Folkman and Shing, 1992; Compagni and Christofori, 2000). Tumour growth and metastasis are dependent on new blood vessel formation (Kim *et al*, 1993; Folkman, 1995). The vessels provide nutrients and a route by which tumour cells exit the primary site and enter the blood stream (Zetter, 1998). Among the various angiogenic stimulators, an important factor involved in normal and pathological angiogenesis is vascular endothelial growth factor (VEGF) (Ferrara, 1999b; Carmeliet and Collen, 2000) which is able to stimulate vascular permeability and is an endothelial cell specific mitogen (Connolly *et al*, 1989). By alternative splicing of a single gene, several VEGF isoforms can be produced (Shima *et al*, 1996). VEGF is secreted by many tumour cell lines *in vitro* and its mRNA is upregulated in the vast majority of human tumours (Ferrara, 1999a).

To investigate the importance and the underlying mechanisms of angiogenesis in the development of MM, an *in vivo* model is required. Therefore we selected the murine 5TMM models. We have used two MM models, which initially originated spontaneously in ageing C57BL/KaLwRij mice (Radl *et al*, 1979). Tumour cells from diseased mice were isolated from the bone marrow and transplanted into young syngeneic recipients by intravenous injection. By this way, several 5TMM models were developed (Radl *et al*, 1979) with similar characteristics as the human disease: the disease occurs spontaneously at older age, the MM cells are localised in the bone marrow, tumour load can be assessed by paraproteinaemia, osteolytic lesions develop and the molecular mechanisms are like in humans (Vanderkerken *et al*, 1996; Radl *et al*, 1988; Asosingh *et al*, 2000). As previously reported, the 5T2MM model more closely represents human MM, characterized by a moderate progressive course of the disease and induction of osteolytic lesions, while the 5T33MM model represents an aggressive, rapidly progressive variant (Vanderkerken *et al*, 1997; Radl *et al*, 1988). Therefore, the 5T2MM and 5T33MM models are good *in vivo* murine models of myeloma which mimic different aspects of the human disease (Asosingh *et al*, 2000).

The aim of this study was to study the ability of 5T2MM and 5T33MM cells to induce angiogenesis in the bone marrow, as do human MM cells. The angiogenic potential of 5T2MM and 5T33MM cells was assessed both *in vitro,* in the aortic ring assay, and *in vivo,* by determining the microvessel density in bone marrow samples of control mice and 5T2MM and 5T33MM-diseased mice. The understanding of neovascularization mechanisms in MM may provide further benefits for both diagnosis and therapy.

## MATERIALS AND METHODS

### Mice

C57BL/KaLwRij mice were purchased from Harlan CPB (Horst, The Netherlands). They were housed and treated following the conditions approved by the Ethical Committee for Animal Experiments, VUB (License no. LA1230281). The animal ethics meet the standards required by the UKCCCR Guidelines (UKCCCR, 1998).

### 5TMM models

The *in vivo* growing 5T2MM and 5T33MM originated initially spontaneous in ageing C57BL/KaLwRij mice and have since been propagated into young syngeneic recipients by intravenous transfer of the diseased bone marrow (Radl *et al*, 1979). The tumour take was followed up by protein electrophoresis of the serum samples (Vanderkerken *et al*, 1997). Mice were killed when a serum paraprotein concentration of 10 mg ml^−1^ was reached. The MM cells in the bone marrow were isolated and purified as previously described (Vanderkerken *et al*, 1999). Bone marrow was flushed from the hind legs. The isolated cells were suspended in RPMI-1640 medium (Gibco, Life Technologies, Gent, Belgium) supplemented with penicillin/streptomycin, glutamine, MEM and sodium pyruvate (Gibco). After washing, mononuclear cells were isolated by Lympholyte M (Cedarlane, Hornby, Canada) gradient centrifugation at 1000 **g** for 20 min. Subsequently, the cells were further purified on a Percoll (Pharmacia, Uppsala, Sweden) 60% (for 5T2) and 70% (for 5T8); gradient centrifugation at 450 **g** for 25 min. Cells were stained with anti-idiotype antibodies and purity was assessed by flow cytometry (Vanderkerken *et al*, 1997). The 5TMM cells (1×10^6^ cells ml^−1^) were cultured in serum-free MCDB131 medium (an optimised medium for microvascular endothelial cell growth) or were immediately snap-frozen for RNA isolation. After 48 h, conditioned media were collected.

### Assessment of angiogenesis by rat aortic ring assay

Angiogenesis was studied by culturing aortic explants in three-dimensional matrix gels according to the procedure of Nicosia and Ottinetti (1990). Thoracic aortas removed from 8- to 12-week-old male Fisher-344 rats were immediately transferred to a culture dish containing cold serum-free Minimum Essential Medium (MEM, Life Technologies). The periaortic fibroadipose tissue was carefully removed with fine microdissecting forceps and iridectomy scissors paying special attention not to damage the aortic wall. Aortic rings (1 mm-long) (approximately 20 per aorta) were sectioned and extensively rinsed in five consecutive washes of MEM medium. Ring-shaped explants of rat aorta were then embedded in gels of rat tail interstitial collagen (1.5 mg ml^−1^) as previously described by Montesano *et al* (1983). The final collagen solution was obtained by mixing 7.5 volumes of collagen (2 mg ml^−1^) (Collagen R, Serva, Heidelberg, Germany) with one volume of 10×MEM, 1.5 volumes of NaHCO_3_ (15.6 mg ml^−1^) and approximately 0.1 volume NaOH (1 M) to adjust the pH to 7.4. The collagen gel cultures were first prepared in cylindrical agarose wells (Nicosia and Ottinetti, 1990) and kept in triplicates at 37°C in 100 mm diameter Petri dishes (bacteriological polystyrene, Falcon, Becton Dickinson, Lincoln Park, NJ, USA). Each dish contained either 30 ml of MCDB131 (Life Technologies) supplemented with 25 mM NaHCO_3_, 1% glutamine, 100 U ml^−1^ penicillin and 100 μg ml^−1^ streptomycin or MCDB131 medium conditioned by 5T2MM or 5T33MM cells. As positive control human recombinant VEGF165 (20 ng ml^−1^) was added to the medium. The cultures were kept at 37°C in a humidified environment for 1 week and examined by phase contrast microscopy with an Olympus microscope at the appropriate magnification. Image analysis was performed on a WorkStation Sun SPARC30, using the software ‘Visilog5.0’ from Noesis. We used an improved computer-assisted image analysis (Blacher *et al*, 2001) which allows automatic measurements of the geometrical and morphological parameters. After generation of binary image, the following automatic measurements were performed: the quantification of microvessels according to the vascular complexity, i.e. the determination of the microvessel distribution given by the total number of intersections of microvessels (N_i_) in function to the distance to the aortic ring. The N_i_ value at the first step of the grid gives the number of microvessels (N_v_). The grid step at which the last N_i_≠0 is measured gives the maximal microvessel length (L_max_). The sum of additional intersections detected at each step of the grid gives the total number of branching (N_b_). Statistical parameters have been calculated to describe the distribution of N_i_. The mean distribution (D) and the standard deviation correspond to the distance from the explant at which N_i_ reaches its mean value. The mode (d) corresponds to the distance from the explant at which most cells migrated. The skewness (sk) describes the symmetry of the distribution and the kurtosis (k) describes how flat or peaked the distribution is.

### Assessment of angiogenesis by bone marrow microvessel density

Long bones, vertebrae and ribs of age-matched control, 5T2MM and 5T33MM diseased C57BL/KaLwRij mice were fixed in zinc fixative (0.1 M Tris buffer, pH 7.4, 3 mM calcium acetate, 0.027 M zinc acetate and 0.037 M zinc chloride) for 24 h and decalcified in FE10 (0.27 M EDTA, 0.3 M NaOH, 4% formalin) for 3 days. The material was embedded in paraffin and 5 μm longitudinal sections throughout the whole bones were made with a rotation microtome (Microm HM335, Germany). One slide was stained with haematoxylin and eosin, the consecutive slide and two additional slides with a 50 μm distance from each other were immunostained for CD31 in order to visualise the blood vessels and sinusoids in the bone marrow.

For immunostaining, the endogenous peroxydase was quenched by incubating the slides with a 50 ml methanol, 0.5 ml H_2_O_2_ (30%) solution for 30 min. Trypsinisation (Trypsin 109827, Boehringer Mannheim, IN, USA) for 20 min, at 37°C was used for antigen retrieval. The slides were preincubated with normal goat serum for 30 min. The primary antibody (PECAM-1, Becton Dickinson PharMingen, LA, USA) was incubated overnight at 4°C at a dilution of 1 out of 100. As secondary antibody, a biotin-conjugated goat anti-rat specific polyclonal immunoglobulin (554014, Becton Dickinson PharMingen, LA, USA) was used at a dilution of 1 out of 100. The TSA (Tyramide Signal Amplification) (NEN Life Science Products, Boston, MA, USA) was used to enhance the signal intensity. Chromogenic visualisation was accomplished through the use of a streptavidin-horseradish peroxydase conjugate, followed by diaminobenzidin.

The haematoxylin and eosin sections of the bone marrow were screened for the presence of myeloma cells, with a light microscope (Axiolab, CarlZeiss, Germany). For quantification of angiogenesis, areas with the highest density of blood vessels (hot spot) were selected on sections immunostained for CD31. The number of blood vessels was counted per 0.22 mm^2^ using a microscope eyepiece graticule (×10 ocular and ×20 objective). The microvessel density was independently evaluated by two observers.

### Detection of mRNA for VEGF

Total RNA from 5×10^6^ purified 5T2MM and 5T33MM cells was isolated using the SV total RNA isolation system (Promega Corporation, Madison WI, USA) according to the manufacturer's instructions. The concentration and purity of RNA was determined by spectrophotometric measurement (Gene Quant II, Pharmacia Biotech, Cambridge, UK).

One to 5 μg of total RNA was converted into cDNA by the superscript first-strand synthesis system (Gibco, Life Technologies) using random hexamers as primers. The cDNA was used as template for PCR in a 25 μl reaction containing: PCR buffer, 2 mM magnesium chloride, 0.2 mM dNTP, 0.5 μM of each primer and 0.625 U Taq polymerase. The sense and antisense primers used for the amplification of the known splice variants of mouse VEGF are 5′-CCTGGTGGACATCTTCCAGGAGTA-3′ and 5′-CTCACCGCCTCGGCTTGTCACA-3′, respectively. Forty PCR cycles were performed preceded by a denaturation step at 95°C for 2 min followed by 20 s at 94°C, 20 s at 66°C and 30 s at 72°C. The predicted size of the amplified products was 479 bp for VEGF188, 407 bp for VEGF164 and 275 bp for VEGF120 (Hajitou *et al,* 2001). PCR products were analysed by electrophoresis on a 1.5% low-melting agarose gel. The PCR samples were cloned into the pCRII vector (Invitrogen, San Diego, CA, USA) and sequenced with vector-specific primers using the Sequenase version 2.0 DNA sequencing kit (USB Corporation, Cleveland, OH, USA).

### Detection of VEGF protein

The secretion of VEGF by the 5TMM cell lines in serum-free conditioned media (48 h) was quantified by an enzyme-linked immunosorbent assay (ELISA, R&Dsystems, Wiesbaden, Germany) according to the manufacturer's instructions. Each sample was performed in duplicate and two independent experiments were done.

### Statistical analysis

For statistical analysis the unpaired student's *t*-test was used. *P*-values<0.05 were considered as significant.

## RESULTS

### 5TMM cells induce angiogenesis *in vitro*

**Figure 1:**
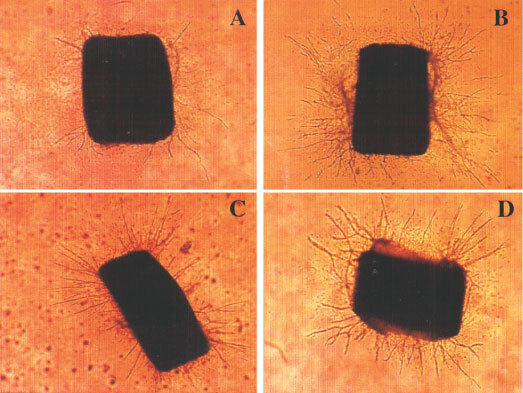
Photomicrographs of rat aortic rings cultured in collagen gels in the presence of serum-free medium (**A**), serum-free medium supplemented with 20 ng ml^−1^ rVEGF165 (**B**), 5T2MM conditioned medium (**C**), 5T33MM conditioned medium (**D**). Photomicrographs from one experiment, representative of there, are shown.

**Figure 2:**
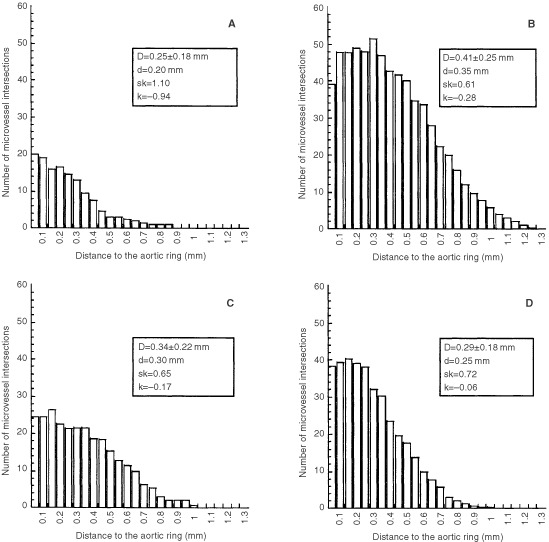
Determination of the number of microvessel intersections (N_i_) in function of the distance to the aortic ring. Histograms have been constructed for control (**A**), VEGF (**B**), 5T2MM (**C**), 5T33MM (**D**). Statistical parameters are indicated for each histogram. D: distance from the explant at which N_i_ reaches its mean value; d: distance from the explant at which most cells migrated; sk>0 indicates that most cells migrate at distances lower than D; k: sharpness of the cell distribution around d.

The angiogenic potential of the 5T2MM and 5T33MM cells was tested in the *in vitro* rat aortic ring assay. Aortic rings were cultured in the presence of serum-free MCDB131 medium or MCDB131 medium conditioned by 5T2MM or 5T33MM cells. As positive control human recombinant VEGF165 was added to the medium. After 1 week, the rings cultured in the presence of 5T2MM (Figure 1C

) or 5T33MM (Figure 1D) conditioned media (Figure 1A) exhibit more capillaries than those cultured in control medium. Interestingly, in the presence of 5T33MM conditioned medium, the total number of microvessels (N_v_=38) was two-fold higher than in control conditions (N_v_=20). The addition of 5T2MM conditioned medium induced a less pronounced, but still statistically significant effect on microvessel outgrowth (N_v_=24). In the presence of both media conditioned by myeloma cells, the maximal microvessel length was 20% enhanced (L_max_=1.0 mm *vs* 0.85 mm in control conditions). The vascular network complexity was more precisely studied by statistical analysis according to the methodology described by Blacher *et al* (2001) (Figure 2

). The broadness of the histogram obtained with both conditioned media indicates that microvessel networks were more branched than in control conditions. Indeed, the N_i_ distribution of 5T2MM and 5T33MM condition had a peak (k ≅ 0) nearby the aortic ring and decreased less steeply (sk=0.65 for 5T2MM and 0.72 for 5T33MM) than in control conditions (sk=1.10). This indicates that myeloma conditioned media enhanced the vascular network complexity which are then characterized by more branched vessels (Figure 2A).

### Assessment of microvessel density in the bone marrow

Using an eye-piece graticule the area with the highest density of blood vessels was selected, discarding the area near the growth plate since this area, as distinctly observed in the non-invaded marrow, has physiologically a higher blood vessel density. When compared to normal bone marrow, the sinusoids were more compressed (probably a mechanical effect) in the tumour-invaded marrows. When dilated sinusoids were observed in invaded marrows, they were massively filled with tumour cells. Since the lumen of the sinusoids belongs to the extramedullary compartment this finding confirms the presence of circulating tumour cells. As previously observed (Vanderkerken *et al*, 1997), the 5T33MM cells were more blastic (centrally located nuclei, distinctive nucleoli) compared to 5T2MM cells in which the plasmacytic differentiation was more readily observed (eccentric location of the nucleus, clumped chromatin).

**Figure 3:**
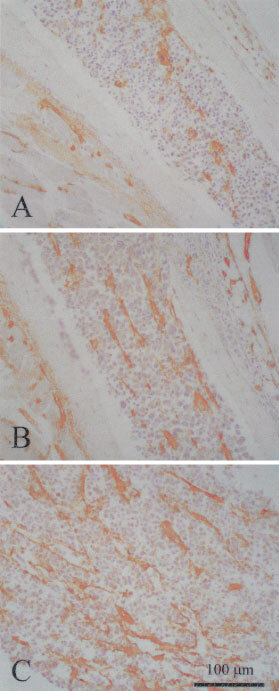
CD31 immunostaining of bone marrow from a rib of a control mouse (**A**), from a rib of a 5T2MM diseased mouse (**B**) and from a femur of a 5T33MM diseased mouse (**C**).

In both 5T2MM and 5T33MM invaded bone marrows, we observed a statistically significant increase in blood vessel density as compared to non-invaded controls (Figures 3

and 4

**Figure 4:**
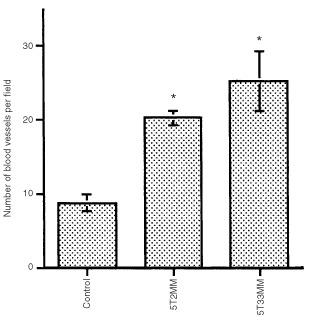
Number of blood vessels counted in the bone marrow of control mice, 5T2MM and 5T33MM diseased mice. The blood vessels were visualised by an immunostaining for CD31. Mean±s.e. values of 14 animals per group are shown. *Indicates significant difference (*P*<0.001) compared with control.

). The hot spots coincided with sites of massive invasion.

### Expression and secretion of VEGF by the 5T2MM and 5T33MM cells

**Figure 5:**
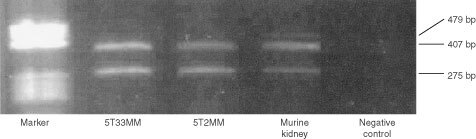
Analysis of VEGF expression in the 5TMM cells by RT–PCR. cDNA from purified myeloma cells were subjected to specific VEGF amplification. The amplification products were detected as a band of 275 bp for VEGF120, 407 bp for VEGF164 and 479 bp for VEGF188. Murine kidney was used as positive control for the three isoforms of VEGF. This experiment is representative for two experiments.

Since VEGF is one of the most important pro-angiogenic factors, we investigated whether 5TMM cells secrete VEGF. The expression of VEGF transcripts was determined by RT–PCR (Figure 5

). The positive control (murine kidney) expresses the three VEGF isoforms. The 5T2MM and 5T33MM cells express VEGF transcripts corresponding to the two lower molecular weight isoforms, VEGF164 and VEGF120. This was confirmed by sequencing the two transcripts (Claffey *et al*, 1992; Shima *et al*, 1996). The secretion of VEGF by both cell lines was further assessed by ELISA. The concentration of VEGF in culture supernatants of the 5T2MM and 5T33MM cells ranged between 100 and 160 pg ml^−1^.

## DISCUSSION

Recently, it was postulated that neovascularization may play an important role in haematological malignancies (Bertolini *et al*, 2000; Mangi and Newland, 2000). It was suggested that, like in solid tumours, the progression of MM takes place in two phases. Nonactive MM and monoclonal gammopathies of undetermined significance (MGUS) might represent the prevascular phase, whereas active MM might correspond to the vascular phase (Vacca *et al*, 1994). Angiogenesis is a complex multistep process characterized first by the stimulation of endothelial cells by angiogenic factors, and then by the production of proteolytic enzymes, such as matrix metalloproteinases (MMPs) and urokinase plasminogen activator (uPA). These enzymes are involved in the degradation of the basement membrane and the extracellular matrix, and promote the migration and proliferation of endothelial cells to form a new blood vessel (Moses, 1997; Zetter, 1998). In human MM, VEGF and bFGF are secreted by tumour cells and the concentrations of these angiogenic factors are higher in more advanced disease (Vacca *et al*, 1998; Bellamy *et al*, 1999; Dankbar *et al,* 2000; Di Raimondo *et al*, 2000; Sezer *et al*, 2000; Van Riet *et al*, 2000). MM cells also secrete MMPs (Barillé *et al*, 1997, 1999; Vanderkerken *et al*, 2000) and uPA (Hjertner *et al*, 2000) and their conditioned medium is able to stimulate the proliferation and migration of endothelial cells (Vacca *et al*, 1998, 1999). These findings suggest that the MM cells can induce angiogenesis. The ability of medium conditioned by plasma cells from MM patients to stimulate neovascularisation in the chorioallantoic membrane assay supports this hypothesis (Vacca *et al*, 1998, 1999). These recent insights disclose new possible therapeutic targets. Interestingly, the anti-angiogenic drug, thalidomide, has been reported to be effective in patients with refractory MM (D'Amato *et al*, 1994; Singhal *et al*, 1999). The development of drugs acting on different levels of angiogenesis can thus be considered as a promising pathway. This research inevitably requires good *in vitro* and *in vivo* experimental models. In the present study, we demonstrate that the 5T2MM and 5T33MM murine multiple myeloma models are interesting *in vivo* models of angiogenesis. Both cell lines are able to induce *in vitro* and *in vivo* angiogenesis. This could be, at least in part, related to their capacity to produce VEGF as demonstrated by RT–PCR and ELISA. These data indicate that MM cells have, at least, the potential to induce the first step of neovascularisation, namely the stimulation of endothelial cells.

The ability of both 5T2MM and 5T33MM cells to induce *in vitro* angiogenesis was measured in the rat aortic ring assay. The microvessels branched, anastomosed, developed lumina and formed complex networks as initially described by Nicosia and Ottinetti (1990). Compared to control condition, a more complex microvessel outgrowth occurred when the rings were cultured in the presence of media conditioned by 5T2MM or 5T33MM cells. However, the network of blood vessels was less complex than the one induced by recombinant VEGF, as assessed by our original method of vessel quantification based on computer-assisted image analysis (Blacher *et al*, 2001). In the rat aortic ring assay a dose-dependent effect of VEGF on microvascular network complexity was evidenced by Blacher *et al* (2001). The much lower VEGF concentration in 5T2MM and 5T33MM conditioned media than the concentration of recombinant VEGF added to the medium of the positive control can explain the difference in network complexity. The MVD in the bone marrow from diseased and control animals was compared in order to investigate the *in vivo* angiogenesis. To achieve a reliable counting of the bone marrow blood vessels several factors had to be fulfilled: representative samples, optimal fixation and decalcification, thin sections and a sensitive immunohistochemical staining of the blood vessels. Whole bones were collected at different sites of the skeleton; fixation and decalcification methods were optimised. Immunostaining for the CD31 antigen of the blood vessels in the BM of 5T2MM and 5T33MM diseased mice revealed a significantly higher MVD in the diseased animals compared to the control animals. The higher angiogenic potential of the 5T33MM model can be due to the more aggressive growth of the 5T33MM cells compared to the 5T2MM cells (Vanderkerken *et al*, 1997).

Based on their *in vivo* and *in vitro* angiogenic potential and their capacity to produce VEGF, we conclude that the two 5TMM models are suitable for the study of multiple myeloma neovascularization. These models can now be used to unravel the multistep process of neovascularization and to test new angiogenic inhibitors.
